# Loss of function mutations in the progranulin gene are related to pro-inflammatory cytokine dysregulation in frontotemporal lobar degeneration patients

**DOI:** 10.1186/1742-2094-8-65

**Published:** 2011-06-06

**Authors:** Paola Bossù, Francesca Salani, Antonella Alberici, Silvana Archetti, Giuseppe Bellelli, Daniela Galimberti, Elio Scarpini, Gianfranco Spalletta, Carlo Caltagirone, Alessandro Padovani, Barbara Borroni

**Affiliations:** 1 IRCCS Santa Lucia Foundation, Via Ardeatina 306, 00179 Rome, Italy; 2 Centre for Neurodegenerative Disorders, Neurology Unit, University of Brescia, Italy; 3 III Laboratory and Biotechnology Department Diagnostic of Laboratories, Brescia Hospital, Brescia, Italy; 4 Department of Internal Medicine, University of Milan Bicocca, Italy; 5 University of Milan, Fondazione Cà Granda, IRCCS Ospedale Maggiore Policlinico, Milan, Italy

## Abstract

The progranulin gene (*PGRN*) encodes a pleiotropic molecule with anti-inflammatory actions and neuronal protective effects. Accordingly, PGRN-deficient mice have been demonstrated to develop enhanced inflammation and progressive neurodegeneration. Loss of function mutations of the *PGRN *gene have been also reported to cause frontotemporal lobar degeneration (FTLD), a neurodegenerative disease leading to dementia generally in the presenium. Since neurodegeneration might be negatively impacted by chronic inflammation, the possible influence of *PGRN *defects on inflammatory pathways appears to be of great relevance for the understanding of neurodegeneration pathogenic processes in those patients. However, no data about the inflammatory profile of *PGRN*-defective subjects have been so far provided.

In this study, we analyzed serum levels of the pro-inflammatory mediators IL-6, TNF-α and IL-18 in FTLD patients with or without *PGRN *mutations, at both pre-symptomatic and symptomatic stages. We provide evidence that circulating IL-6 is increased in *PGRN*-mutated FTLD patients, as compared to both *PGRN *non-mutated FTLD patients and controls. In contrast, levels of IL-6 were not altered in asymptomatic subjects carrying the *PGRN *mutations. Finally, TNF-α and IL-18 serum levels did not differ among all groups of included subjects.

We conclude that the profile of circulating pro-inflammatory cytokines is altered in *PGRN-*related symptomatic FTLD. Thus, our findings point to IL-6 as a possible specific mediator and a potential therapeutic target in this monogenic disease, suggesting that an enhanced inflammatory response might be indeed involved in its progression.

## Introduction

Progranulin (PGRN), also known as granulin-epithelin precursor, is a pleiotropic molecule promoting survival, cell cycle progression, proliferation, and migration of several cell types, and is recognized as an important regulator of the wound-healing response [1,2]. In the periphery, PGRN is involved in inflammation regulation and, as a whole molecule, it possesses anti-inflammatory effects [3,4]. Within the brain, PGRN is up-regulated in neuroinflammatory conditions and its increased expression by microglia may be relevant in responses to brain injury, neuroinflammation and neurodegeneration [5,6]. PGRN is encoded by a single gene on chromosome 17q21 and, interestingly, loss of-function mutations in the *PGRN *gene have been identified as a monogenic cause of frontotemporal lobar degeneration (FTLD) [7-9]. Up to now, almost 70 pathogenic *PGRN *mutations have been described, and all are expected to cause PGRN haploinsufficiency (Alzheimer Disease and Frontotemporal Dementia Database. http://www.molgen.ua.ac.be/FTDmutations/). Patients with *PGRN *mutations may present with initial disease symptoms typically in their 50s or 60s, with a wide spectrum of disease onset and clinical presentation [10].

The heterogeneity of *PGRN-*related FTLD suggests that several other factors of both genetic and environmental nature, including inflammation, can influence disease expression. Indeed, similar to other neurodegenerative disorders, inflammatory mechanisms might participate in the pathophysiology of FTLD, since the disease is characterized by *in vivo *activation of microglia [11], and increased levels of intrathecal pro-inflammatory mediators [12,13]. However, no data in FTLD patients establishing a link between *PGRN *mutations and inflammatory changes have been so far provided, and demonstration of an imbalance in inflammation pathways and establishment of the timing of inflammation abnormalities in *PGRN*-related disease might be of crucial help in identifying new therapeutic targets for this orphan disorder.

In the present study, we evaluated FTLD patients with and without PGRN haploinsufficiency for circulating levels of cytokines with pro-inflammatory action that are likely relevant to neurodegeneration, epitomized by IL-6, TNF-α and IL-18. In particular, IL-6 and TNF-α, which are measurable in human serum, were chosen here as inflammatory markers because of their implication in the animal model of PGRN deficiency-mediated neurodegeneration [14]. In addition, the cytokine IL-18 was also included in this study because of its pivotal role in neuroinflammation and its association with cognitive decline in demented patients with Alzheimer's disease [15]. Finally, asymptomatic *PGRN *mutation carriers were evaluated to assess the inflammatory cytokine profile in pre-symptomatic disease stages.

## Materials and methods

Patients were recruited who met clinical criteria for FTLD [16,17]. All subjects underwent a somatic and neurological evaluation, routine laboratory tests, a brain structural imaging study, and blood sampling for genetic screening and serum PGRN dosage. The diagnostic assessment was accomplished by a review of full medical history, a semi-structured neurological examination, and a complete standardized neuropsychological status evaluation. Inclusion and exclusion criteria were applied as previously reported [18].

A group of healthy controls (HC) were further included. They were neither related to one another nor to FTLD patients; in particular, we selected only subjects in the same patients' age range, with a MMSE score ≥26 and a formal neurological examination that excluded the presence of a dementing condition.

Moreover, siblings belonging to families of FTLD patients bearing *PGRN *mutations were considered. Cognitive or behavioral deficits were excluded by a standardized neuropsychological assessment. These were subgrouped into asymptomatic subjects carrying *PGRN *mutation (ASYMP-*PGRN+*), to assess inflammatory profiles before *PGRN*-related FTLD onset, and those without *PGRN *mutations (ASYMP-*PGRN-*). A control group similar in age composition was recruited as well.

The study was performed with the understanding and written consent of each subject, and was reviewed and approved by the local ethical committee.

Venous blood samples were drawn from each patient for *PGRN *sequencing. Total genomic DNA was prepared from peripheral blood according to standard procedures, as previously published [9]. All the 12 exons plus exon 0 of *PGRN *and at least 30 base pairs (bp) of their flanking introns were sequenced. Serum PGRN was assessed by enzyme-linked immunosorbent assay (ELISA) kit (AdipoGen Inc), according to the manufacturer's instructions. Similarly, IL-6 and TNF-α were measured using specific high-sensitivity ELISA kits (Quantikine HS, R&DSystems), while IL-18 was determined using antibodies (coating: clone 125-2H; detecting: clone 159-12B) and standards from MBL (Nagoya, Japan). The limit of detection for these assays was 0.16 pg/ml for IL-6; 0.5 pg/ml for TNF-α and 15 pg/ml for IL-18. Comparisons among the subject groups on continuous variables were performed using univariate analyses of variance. Correlations between cytokine serum levels and demographic or clinical characteristics were performed by using correlation analyses (Pearson's r) and Fisher's r to z transformation. The level of statistical significance was set at *P *< 0.05.

## Results

### Serum cytokine levels in FTLD patients and control subjects

Ninety-two FTLD patients, namely 78 without *PGRN *mutations (i.e. FTLD *PGRN*-) and 14 carrying *PGRN *mutations (FTLD *PGRN*+; g.1977_1980delCACT, n = 13 and g.2473C > T, n = 1) along with 59 healthy age-matched controls were included in the present study.

The socio-demographic, clinical and biochemical data about the FTLD patients and the age-matched control subjects are summarized in Table 1. Cytokine levels were analyzed in serum of all subjects and expressed as mean pg/ml ± SEM.

**Table 1:** Characteristics of subjects included in the study

	Healthy controls	FTLD	
		FTLD *PGRN*-	FTD *PGRN*+
Total number	59	78	14
Gender (female), %	57	44	60
Age, years	65.2 ± 1.0	65.5 ± 0.8	63.3 ± 2.3
Diagnosis, bvFTD/PPA, %	-	75.6/24.4	71.4/28.6
MMSE, score	29.1 ± 0.13	21.3 ± 0.8*	18.5 ± 3.0*
Progranulin, ng/ml	n.a.	179.7 ± 6.6	63.4 ± 9.2**

FTLD: frontotemporal lobar degeneration; *PGRN*-: no progranulin pathogenetic mutations; *PGRN*+: progranulin pathogenetic mutations.

Results are expressed as mean ± SEM (standard error), or otherwise specified. n.a.: not available

* *P *< 0.0001, significantly different from healthy control subjects

** *P *< 0.0001, significantly different from FTLD

**Figure 1:**
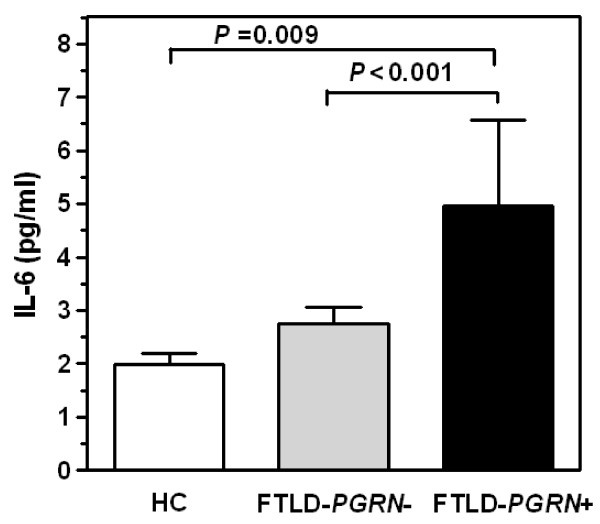
**Serum IL-6 levels in FTLD patients and control subjects**. IL-6 levels in FTLD patients carrying (FTLD *PGRN*+, black bar) and not carrying (FTLD *PGRN*-, grey bar) progranulin mutations and age-matched healthy controls (HC, open bar). Results are reported as histograms representing IL-6 pg/ml mean values with SEM error bars.

As reported in Figure 1, in FTLD *PGRN*+, serum IL-6 levels were significantly increased (4.95 ± 1.61) as compared to FTLD *PGRN*- (2.74 ± 0.31, *P *< 0.001) and HC (2.00 ± 0.20, *P *= 0.009).

TNF-α did not significantly differ in FTLD *PGRN*+ (1.33 ± 0.79) as compared to FTLD *PGRN*- (3.17 ± 5.80, *P *= 0.656) and HC (2.14 ± 1.54, *P *= 0.201).

Comparably, no differences in IL-18 levels were observed in FTLD *PGRN*+ (443.7 ± 168.6), than FTLD *PGRN*- (414.2 ± 139.1, *P *= 0.582) and HC (433.3 ± 178.9, *P *= 0.848).

There were no significant correlations between levels of the three cytokines analyzed and cognitive impairment, as measured by Clinical Dementia Rating for Frontotemporal Dementia and Mini-Mental State Examination, in FTLD patients, regardless their *PGRN *mutation status (data not shown).

### Serum cytokine levels in asymptomatic patients with PGRN mutations and control subjects

In order to verify whether increased levels of IL-6 detected in FTLD with *PGRN *mutations were present before the development of the disease, we analyzed 11 ASYMP *PGRN+ *(g.1977_1980delCACT). At the time of recruitment, these subjects belonged to the second generation of nuclear families, in which the case indexes showed a mean disease onset at 63.3 ± 2.3 years. Based on these demographic data, we are allowed to consider that ASYMP *PGRN+ *subjects, with a mean age of 42.9 ± 4.6 years, were enrolled in a period of their life almost 20 years before the estimated onset of symptoms. Eighteen young healthy subject controls and 14 ASYMP *PGRN- *young asymptomatic controls with no pathogenic *PGRN *mutations were included in the study as well. Demographic characteristics of the 3 groups are shown in Table 2.

**Table 2:** Characteristics of asymptomatic subjects included in the study

	Young healthy controls	Asymptomatic subjects	
		ASYMP-*PGRN-*	ASYMP-*PGRN+*
Total number	18	14	11
Gender (females %)	61.1	57.1	41.7
Age, years (mean ± SEM)	42.2 ± 3.1	43.7 ± 3.2	42.9 ± 4.6
MMSE, score (mean ± SEM)	29.7 ± 0.2	30 ± 0.0	29.25 ± 0.5
Progranulin, ng/ml (mean ± SEM)	n.a.	204.3 ± 25.5	58.6 ± 17.4**

** *P *< 0.0001, significantly different from ASYMP-*PGRN-*.

ASYMP-*PGRN-: *young asymptomatic subjects with no pathogenic progranulin mutations; ASYMP-*PGRN+: *young asymptomatic subjects with pathogenic progranulin mutations.

Different from what was observed in patients with symptomatic FTLD carrying *PGRN *mutations, the levels of IL-6 in patients who had not yet developed the disease were not higher than those of age-matched controls. There were no significant differences between ASYMP *PGRN+ *(1.03 ± 0.73) and ASYMP *PGRN- *(1.53 ± 2.06, *P *= 0.465) or age-matched young controls (1.51 ± 1.88, *P *= 0.459). These data suggest that IL-6 systemic dysregulation in FTLD subjects with PGRN loss may be associated with disease development.

Similar negative results were also obtained for the other two cytokines, i.e. TNF-α and IL-18.

## Discussion

Recent studies on PGRN-deficient mice suggest that endogenous PGRN plays a central role in the regulation of inflammatory cytokine production. In particular, PGRN deficiency causes progressive neurodegeneration through enhanced microglia activation and chronic inflammation characterized by increased production of pro-inflammatory molecules, such as IL-6 and TNF-α [14,19,20]. Indeed, the pleiotropic pro-inflammatory cytokines IL-6 and TNF-α have been proposed to exert both protective and detrimental effects in CNS, but their neurotoxic capability might prevail in chronic conditions [21,22]. In accord with animal data, the main results of our study indicate that the pro-inflammatory cytokine IL-6 is significantly elevated in FTLD *PGRN+ *patients, as a consequence of both the loss of functional PGRN and the development of dementia. In the same direction, a previous study has shown that polymorphisms in the IL-6 gene can modify cognitive and behavioral performances in FTLD patients [23], supporting the potential specific role of this cytokine in influencing symptom occurrence in FTLD. However, in our sample of *PGRN*-mutated and non-mutated FTLD subjects, there was no correlation between serum levels of IL-6 and MMSE scores, suggesting that the relationship between peripheral cytokine elevation and cognitive impairment might be more complex than the association of cytokine gene variants with the disease.

Different from IL-6, in our study TNF-α tends, without reaching statistical significance, to be higher in FTLD patients without *PGRN *mutations, as compared to controls. Indeed, another study reported that in FTLD patients *versus *controls, cerebrospinal fluid levels of TNF-α were significantly increased, without statistically significant changes in circulating TNF-α [13]. Thus, it cannot be excluded that TNF-α might play a role in FTLD, likely independent upon PGRN haploinsufficiency, but this needs to be further investigated. Finally, although emerging data suggest that another pro-inflammatory cytokine, namely IL-18, might be associated with dementia of Alzheimer's type [15], in this study we did not observe any differences in IL-18 serum concentration between FTLD subjects and controls, suggesting that dissimilar inflammatory mechanisms might be implicated in the two different types of dementia.

Overall, these data show for the first time a direct link between an increase in systemic inflammation and PGRN deficiency in FTLD patients, implicating IL-6 as a specific player in this type of FTLD-related inflammatory dysregulation. More specifically, as serum IL-6 concentration has been demonstrated to be unchanged in asymptomatic, preclinical stages of the disease, we can argue that an enhanced IL-6-dependent inflammatory response is crucial for symptom development and progression. Thus, pharmacological inhibition of inflammation, obtained for instance by treating patients with either anti-inflammatory drugs or, ideally, with specific cytokine inhibitors as anti-IL-6 or anti-IL-6R antibody, should be explored as a valuable intervention to slow neurodegeneration in *PGRN*-defective FTLD patients.

This work has some limitations that need to be taken into account for discussion purposes. First of all, the number of subjects, namely patients carrying *PGRN *mutations, is low and the results should be further replicated in larger samples. Moreover, it might be of interest to study an extended panel of cytokines, to study peripheral *versus *intrathecal cytokine modulation, and to study *ex-vivo *cellular production of inflammatory mediators and post-mortem analysis of brain tissue to elucidate in depth the inflammatory response pathway changes that underlay PGRN insufficiency. Finally, although IL-6 apparently exerts a predominantly detrimental action in chronic brain disorders [21], the molecular mechanisms determining IL-6 effects on FTLD neurodegenerative pathways remain to be investigated.

Despite these limitations, this study demonstrates for the first time an increased inflammatory response in *PGRN*-defective humans, suggesting that inflammation might participate in leading to the onset of FTLD symptoms, and highlights possible new molecular targets for *PGRN*-related FTLD treatment.

## List of abbreviations

ASYMP: asymptomatic subjects; ELISA: enzyme-linked immunosorbent assay; HC: healthy controls; FTLD: frontotemporal lobar degeneration; PGRN: progranulin.

## Competing interests

The authors declare that they have no competing interests.

## Authors' contributions

PB and BB designed the study. AA, GB, ES, GS, CC, AP and BB selected patients and performed all clinical evaluations. FS performed cytokine experiments. SA and DG performed the genetic screening. PB, FS and BB analyzed the data. PB and BB wrote the initial draft. All authors made contributions in writing and in discussing the manuscript. All authors have read and approved its final version.

## References

[B1] He Z Bateman A Progranulin (granulin-epithelin precursor, PC-cell-derived growth factor, acrogranin) mediates tissue repair and tumorigenesis J Mol Med 2003 81 600 612 10.1007/s00109-003-0474-3 12928786

[B2] Eriksen JL Mackenzie IR Progranulin: normal function and role in neurodegeneration J Neurochem 2008 104 287 297 17953663 10.1111/j.1471-4159.2007.04968.x

[B3] Zhu J Nathan C Jin W Sim D Ashcroft GS Wahl SM Lacomis L Erdjument-Bromage H Tempst P Wright CD Ding A Conversion of proepithelin to epithelins: roles of SLPI and elastase in host defense and wound repair Cell 2002 111 867 878 10.1016/S0092-8674(02)01141-8 12526812

[B4] Kessenbrock K Frohlich L Sixt M Proteinase 3 and neutrophil elastase enhance inflammation in mice by inactivating anti-inflammatory progranulin J Clin Invest 2008 118 2438 2447 18568075 10.1172/JCI34694 PMC2430496

[B5] Wang X Li X Xu L Zhan Y Yaish-Ohad S Erhardt JA Barone FC Feuerstein GZ Up-regulation of secretory leukocyte protease inhibitor (SLPI) in the brain after ischemic stroke: adenoviral expression of SLPI protects brain from ischemic injury Mol Pharmacol 2003 64 833 840 10.1124/mol.64.4.833 14500739

[B6] Ahmed Z Mackenzie IR Hutton ML Dickson DW Progranulin in frontotemporal lobar degeneration and neuroinflammation J Neuroinflammation 2007 4 7 10.1186/1742-2094-4-7 17291356 PMC1805428

[B7] Baker M Mackenzie IR Pickering-Brown SM Gass J Rademakers R Lindholm C Snowden J Adamson J Sadovnick AD Rollinson S Cannon A Dwosh E Neary D Melquist S Richardson A Dickson D Berger Z Eriksen J Robinson T Zehr C Dickey CA Crook R McGowan E Mann D Boeve B Feldman H Hutton M Mutations in progranulin cause tau-negative frontotemporal dementia linked to chromosome 17 Nature 2006 42 916 919 10.1038/nature05016 16862116

[B8] Cruts M Gijselinck I van der Zee J Engelborghs S Wils H Pirici D Rademakers R Vandenberghe R Dermaut B Martin JJ van Duijn C Peeters K Sciot R Santens P De Pooter T Mattheijssens M Van den Broeck M Cuijt I Vennekens K De Deyn PP Kumar-Singh S Van Broeckhoven C Null mutations in progranulin cause ubiquitinpositive frontotemporal dementia linked to chromosome 17q21 Nature 2006 442 920 924 10.1038/nature05017 16862115

[B9] Borroni B Archetti S Alberici A Agosti C Gennarelli M Bigni B Bonvicini C Ferrari M Bellelli G Galimberti D Scarpini E Di Lorenzo D Caimi L Caltagirone C Di Luca M Padovani A Progranulin genetic variations in frontotemporal lobar degeneration: evidence for low mutation frequency in an Italian clinical series Neurogenetics 2008 9 197 205 10.1007/s10048-008-0127-3 18392865

[B10] van Swieten JC Heutink P Mutations in progranulin (GRN) within the spectrum of clinical and pathological phenotypes of frontotemporal dementia Lancet Neurol 2008 7 965 974 10.1016/S1474-4422(08)70194-7 18771956

[B11] Cagnin A Rossor M Sampson EL Mackinnon T Banati RB In vivo detection of microglial activation in frontotemporal dementia Ann Neurol 2004 56 894 897 10.1002/ana.20332 15562429

[B12] Galimberti D Schoonenboom N Scheltens P Fenoglio C Venturelli E Pijnenburg YA Bresolin N Scarpini E Intrathecal chemokine levels in Alzheimer disease and frontotemporal lobar degeneration Neurology 2006 66 146 147 16401871 10.1212/01.wnl.0000191324.08289.9d

[B13] Sjögren M Folkesson S Blennow K Tarkowski E Increased intrathecal inflammatory activity in frontotemporal dementia: pathophysiological implications J Neurol Neurosurg Psychiatry 2004 75 1107 1111 10.1136/jnnp.2003.019422 15258209 PMC1739153

[B14] Yin F Banerjee R Thomas B Zhou P Qian L Jia T Ma X Ma Y Iadecola C Beal MF Nathan C Ding A Exaggerated inflammation, impaired host defense, and neuropathology in progranulin-deficient mice J Exp Med 2010 207 117 128 10.1084/jem.20091568 20026663 PMC2812536

[B15] Bossù P Ciaramella A Salani F Vanni D Palladino I Caltagirone C Scapigliati G Interleukin-18, from neuroinflammation to Alzheimer's Disease Curr Pharm Design 2010 16 4212 4223 10.2174/138161210794519147 21184660

[B16] Neary D Snowden JS Gustafson L Passant U Stuss D Black S Freedman M Kertesz A Robert PH Albert M Boone K Miller BL Cummings J Benson DF Frontotemporal lobar degeneration: a consensus on clinical diagnostic criteria Neurology 1998 51 1546 1554 9855500 10.1212/wnl.51.6.1546

[B17] McKhann GM Albert MS Grossman M Miller B Dickson D Trojanowski JQ Clinical and Pathological Diagnosis of Frontotemporal Dementia. Report of the work group on frontotemporal dementia and Pick's disease Arch Neurol 2001 58 1803 1809 10.1001/archneur.58.11.1803 11708987

[B18] Borroni B Grassi M Agosti C Paghera B Alberici A Di Luca M Perani D Padovani A Latent profile analysis in frontotemporal lobar degeneration and related disorders: clinical presentation and SPECT functional correlates BMC Neurol 2007 16 7 9 10.1186/1471-2377-7-9 PMC1884173 17506892

[B19] Ahmed Z Sheng H Xu YF Lin WL Innes AE Gass J Yu X Wuertzer CA Hou H Chiba S Yamanouchi K Leissring M Petrucelli L Nishihara M Hutton ML McGowan E Dickson DW Lewis J Accelerated lipofuscinosis and ubiquitination in granulin knockout mice suggest a role for progranulin in successful aging Am J Pathol 2010 177 311 24 10.2353/ajpath.2010.090915 20522652 PMC2893674

[B20] Yin F Dumont M Banerjee R Ma Y Li H Lin MT Beal MF Nathan C Thomas B Ding A Behavioral deficits and progressive neuropathology in progranulin-deficient mice: a mouse model of frontotemporal dementia FASEB J 2010 24 4639 4647 10.1096/fj.10-161471 20667979 PMC2992364

[B21] Spooren A Kolmus K Laureys G Clinckers R De Keyser J Haegeman G Gerlo S Interleukin-6, a mental cytokine Brain Res Rev 2011 in press PubMed PMID: 21238488 10.1016/j.brainresrev.2011.01.002 21238488

[B22] Pan W Zadina JE Harlan RE Weber JT Banks WA Kastin AJ Tumor necrosis factor-alpha: a neuromodulator in the CNS Neurosci Biobehav Rev 1997 21 603 613 10.1016/S0149-7634(96)00047-4 9353794

[B23] Rainero I Rubino E Cappa G Rota E Valfrè W Ferrero P Fenoglio P Baci D D'Amico G Vaula G Gallone S Pinessi Pro-inflammatory cytokine genes influence the clinical features of frontotemporal lobar degeneration Dement Geriatr Cogn Disord 2009 27 543 547 10.1159/000225962 19546559

